# Osseous Metaplasia in Mucinous Tubular and Spindle Cell Carcinoma of the Kidney: A Case of Massive, Bilateral Tumors

**DOI:** 10.1155/2015/465450

**Published:** 2015-08-12

**Authors:** Aeen M. Asghar, Matthew A. Uhlman, Laila Dahmoush, Sundeep Deorah

**Affiliations:** ^1^Carver College of Medicine, University of Iowa, 375 Newton Road, Iowa City, IA 52242, USA; ^2^Department of Urology, University of Iowa, 200 Hawkins Drive, 3 Roy Carver Pavilion, Iowa City, IA 52242-1089, USA; ^3^Department of Pathology, University of Iowa, 200 Hawkins Drive, 5 Roy Carver Pavilion, Iowa City, IA 52242-1089, USA

## Abstract

Renal cell carcinoma (RCC) is the most common kidney malignancy, with many histologic subtypes. One of the rare forms of RCC is mucinous tubular and spindle cell carcinoma (MTSCC), which is newly described with limited information on clinical picture and outcome. Heterotopic bone formation (osseous metaplasia) is a rare finding within any renal mass. Here we report a case of a massive, bilateral MTSCC with histologic findings of heterotopic bone formation, which has not been described before.

## 1. Introduction

In 2014, there were an estimated 63,920 new renal malignancies in the United States, accounting for 13,860 deaths [1]. Renal cell carcinoma (RCC) is the most common primary tumor of the kidney with multiple histological subsets. This includes mucinous tubular and spindle cell carcinoma of the kidney (MTSCC-K), a rare low-grade subtype with rare incidences of metastasis [2]. MTSCC usually presents as a solitary unilateral tumor, in contrast to the patient presenting here with bilateral, massive tumors and heterotopic bone formation. While calcification is seen in about 10% of RCC cases [3], heterotopic bone formation is extremely rare in any subtype of RCC, with only two previous reports in MTSCC-K [4, 5]. The factors leading to heterotopic bone formation are unknown; however, recently, the role of bone morphogenetic protein-2 (BMP-2) has been suggested to both induce osseous bone formation and inhibit tumorigenicity of cancer stem cells [6].

## 2. Case Summary

**Figure 1:**
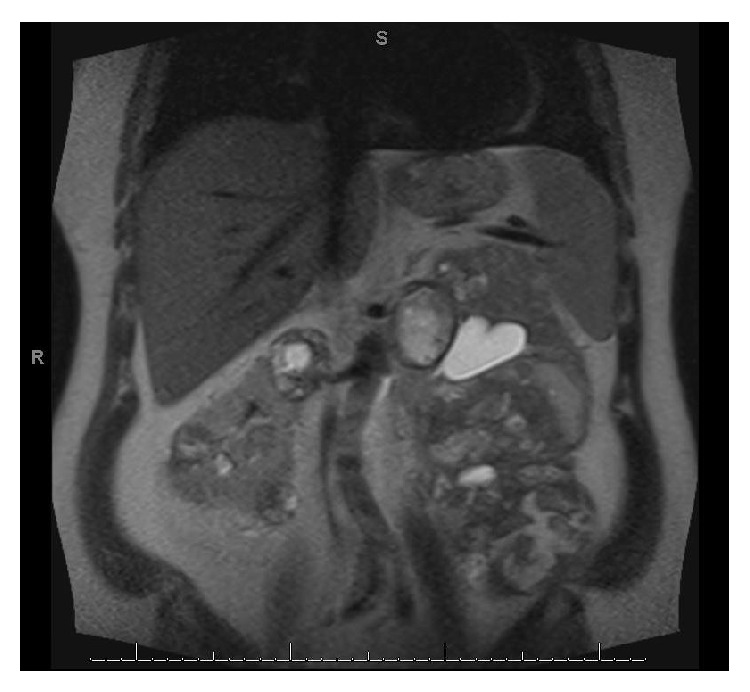
Preoperative abdominal MRI.

A 47-year-old Caucasian male presented to his primary care physician with a one-week history of right upper quadrant abdominal discomfort. He had an estimated GFR of 54 mL/min/1.73 m^2^, but no history of gross hematuria or tobacco use. He has a positive family history for unspecified kidney cancer in his father. Patient's right upper quadrant ultrasound revealed a solid mass measuring 3.5 × 3.7 × 3.4 cm in the right kidney, suspicious for RCC. Abdominal CT scan performed at an outside institution showed multiple nonenhancing mildly hyperdense left renal lesions measuring between 20 and 60 Hounsfield units and right renal nonenhancing cystic lesions measuring between 22 and 38 Hounsfield units, consistent with APKD. However, given the atypical features, an abdominal MRI was performed to further characterize the lesions; the patient underwent MRI of the abdomen which revealed numerous hemorrhagic renal cystic lesions with soft tissue components concerning multifocal renal cell carcinoma in both left and right kidneys; the left side lesions were much larger than the right side ones (Figure 1). Metastatic workup included chest X-ray and was negative.

**Figure 2:**
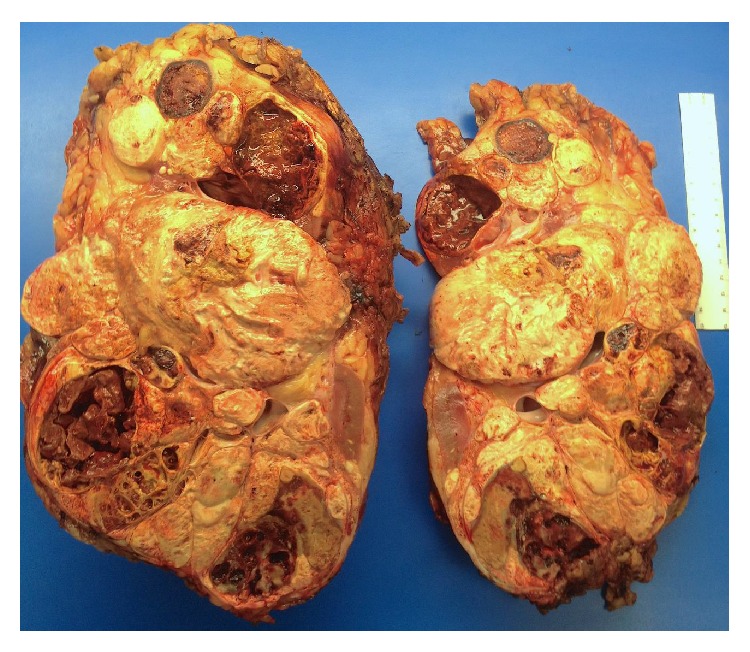
Gross left kidney with mucinous tubular and spindle cell carcinoma.

**Figure 3:**
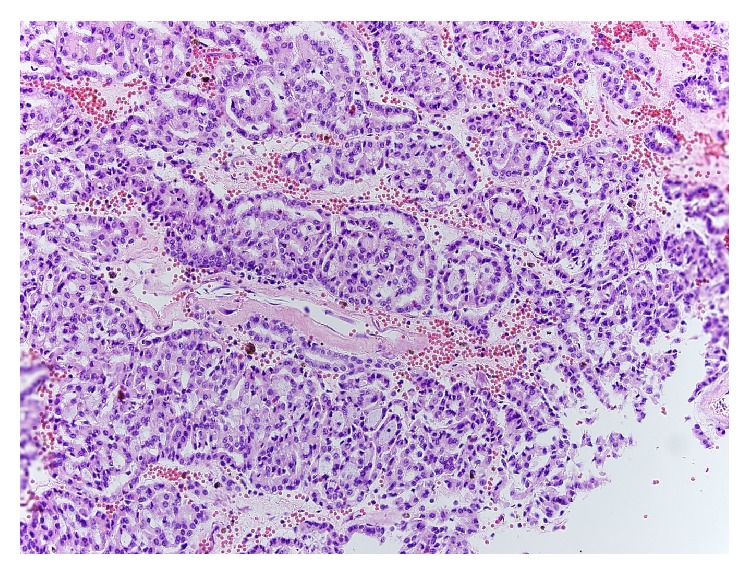
Tumor cells forming merging tubules containing mucin.

**Figure 4:**
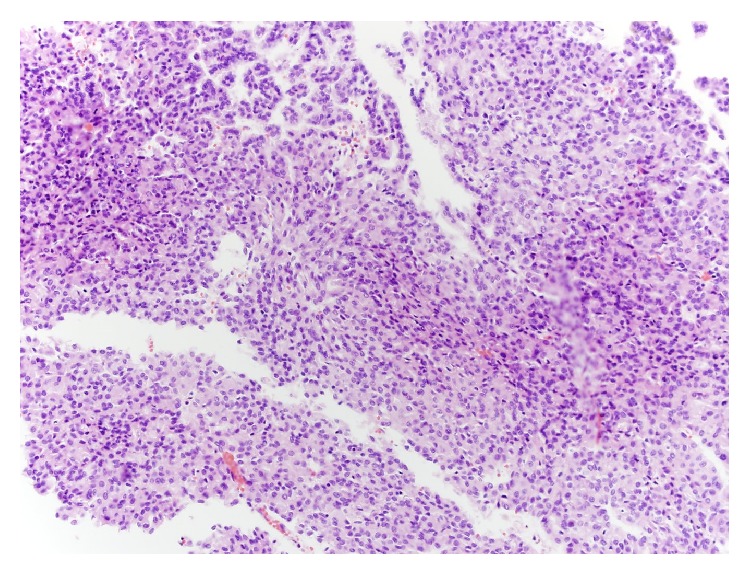
Tumor cells showing spindle cell morphology at the center.

**Figure 5:**
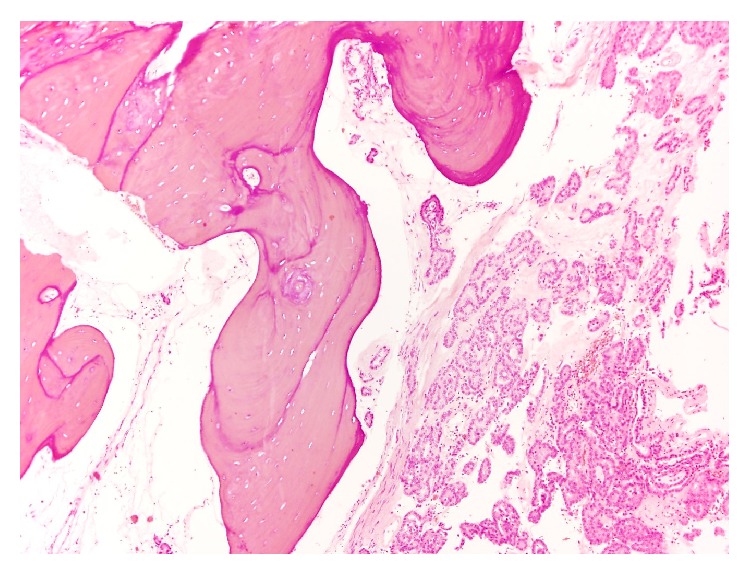
Bone trabeculae are seen adjacent to tumor cells.

Due to the bilateral nature of the masses and atypical appearance, ultrasound guided biopsies of the solid areas of left kidney were performed and revealed MTSCC-K, Fuhrman nuclear grade 2/4. The right-sided lesions were presumed to have similar pathology based upon similar radiological appearance. After discussion of treatment options, the patient opted for a left radical nephrectomy and close monitoring of the right kidney. He underwent left radical nephrectomy uneventfully with surgical pathology demonstrating the mass to be well-circumscribed and measuring 28.8 cm in greatest dimension. Gross pathology revealed well-circumscribed renal mass with multinodular, tan yellow to red brown cut surfaces with focal areas of bone formation (Figure 2). There was sinus fat invasion and surgical margins were negative. Microscopic examination of the tumor showed features characteristic of MTSCC, with some areas showing crowded, merging tubules containing bluish-tinged mucin (Figure 3) and others showing spindle shaped morphology (Figure 4). Final Fuhrman nuclear grade was 3/4. Focal areas of bone formation were also noted (Figure 5). Final tumor staging was T3aNxM0. The patient recovered smoothly from the surgery and his postoperative eGFR is 43 mL/min/1.73 m^2^. At this last visit, he had 3-month surveillance imaging which revealed stable right-sided lesions and no evidence of recurrence in the left nephrectomy bed.

## 3. Discussion

MTSCC is an emerging form of RCC, which was first included in the 2004 WHO classification of renal tumors in adults [7]. With the largest series to date having 19 patients [8], the natural history of this subtype of tumor is not well-established. However, the tumor often presents as a circumscribed mass on ultrasound and is often indolent with extremely rare reports of metastasis to lymph nodes and other organs [2, 9].

The tumor presented in this case is the largest MTSCC described in the literature measuring 28.8 cm in greatest dimension and the only bilateral case of MTSCC in current literature. Despite bilaterally and a large disease burden, the tumor was localized without metastasis, given the indolent nature. The bilaterality of disease raises question of a genetic component, especially with his family history. Patient was offered genetic testing and he declined. Because of relatively small size of right side lesions, we agreed to follow it closely with periodic scans.

Surgical pathology was also able to identify heterotrophic bone formation. While calcified foci are well known and present in up to 10.3% of RCCs [3], ossification is extremely rare [10, 11]. There is currently no clear mechanism of ossification identified; however, since RCCs are known to undergo hemorrhage, necrosis, fibrosis, and hyalinization, it is plausible that osseous metaplasia is secondary to these changes [12].

Another hypothesis centers on dedifferentiation of neoplastic cells into a sarcomatous proliferation [10]. Bone morphogenetic protein-2 (BMP-2), a multifunctional cytokine able to induce bone formation, has recently been described by Wang et al. as being an inhibitor of tumorigenicity in RCC neoplastic stem cells [6]. As such, heterotopic bone formation in this massive tumor may have played a protective role, especially given that the tumor grew to such a large size without invading the surrounding structure or developing distant metastasis. However, another explanation for the nonaggressive behavior of the tumor could be the natural docile behavior of MTSCC. As recombinant human BMP-2 is studied for its role in treatment of RCC [6], it is important to characterize heterotrophic bone formation in all forms of RCC, including MTSCC, and to relate it to the prognosis of patients with RCC.

## 4. Conclusion

MTSCC is a rare subset of renal cell carcinomas with heterotopic bone formation within such a tumor being exceedingly rare. Here we describe the first bilateral MTSCC, which is also the largest one reported and only the third such tumor with heterotopic bone formation. While MTSCC-Ks are generally indolent, the lack of invasion or metastases in a tumor of this size is rather striking. As such, the question of whether or not this may be attributed to the protective role of BMP-2 is very pertinent. Future studies will likely illuminate the potential protective properties of this protein.
